# Performance of new adjustments to the TRISS equation model in developed and developing countries

**DOI:** 10.1186/s13017-017-0129-2

**Published:** 2017-03-28

**Authors:** Cristiane de Alencar Domingues, Raul Coimbra, Renato Sérgio Poggetti, Lilia de Souza Nogueira, Regina Marcia Cardoso Sousa

**Affiliations:** 1 Faculdade das Américas, São Paulo, SP Brazil; 2 University of California San Diego Medical Center, San Diego, CA USA; 3 Medical School, University of Sao Paulo, São Paulo, SP Brazil; 4 School of Nursing, University of Sao Paulo, São Paulo, SP Brazil

**Keywords:** Traumatology, Wounds and injuries, Injury severity score, Outcome assessment

## Abstract

**Background:**

The Trauma and Injury Severity Score (TRISS) has been criticized for being based on data from the USA and Canada—high-income countries—and therefore, it may not be applicable to low-income and middle-income countries. The present study evaluated the accuracy of three adjustments to the TRISS equation model (NTRISS-like; TRISS SpO_2_; NTRISS-like SpO_2_) in a high-income and a middle-income country to compare their performance when derived and applied to different groups.

**Methods:**

This was a retrospective study of trauma patients admitted to two institutions: a university medical center in São Paulo, Brazil (a middle-income country), and a level 1 university trauma center in San Diego, USA (a high-income country). Patients were admitted between January 1, 2006, and December 31, 2010. The subjects were 2416 patients from Brazil and 8172 patients from the USA. All equations had adjusted coefficients for São Paulo and San Diego and for blunt and penetrating trauma. Receiver operating characteristic (ROC) curves were used to evaluate performance of the models.

**Results:**

Regardless of the population where the equation was generated, it performed better when applied to patients in the USA (AUC from 0.911 to 0.982) compared to patients in Brazil (AUC from 0.840 to 0.852). When the severity was considered and homogenized, the performance of equations were similar to both application in the USA and Brazil.

**Conclusions:**

Survival probability models showed better performance when applied in data collected in the high-income countries (HIC) regardless the country they were derived. The severity is an important factor to consider when using non-adjusted survival probability models for the local population. Adjusted models for severely traumatized patients better predict survival probability in less severely traumatized populations. Other factors besides physiological and anatomical data may impact final outcomes and should be identified in each environment if they are to be used in the development of the trauma care performance improvement process in middle-income countries.

## Background

The Trauma and Injury Severity Score (TRISS) methodology is widely accepted in assessing quality of care and promoting improvements in trauma care despite criticism for being derived from a database on trauma victims in the USA and Canada, presenting results and regression coefficients that relate to these countries’ realities [1]. USA and Canada present mature trauma systems, and their results can serve as targets to be achieved by other countries. However, those systems do not reflect the difficulties in providing care faced in other countries due to specific geographic, economic, and sociodemographic characteristics, standards for health care systems, and local morbidity and mortality rates [2].

Trauma quality improvement programs are a fundamental part of trauma care systems in developed countries and have been used in some low-income and middle-income countries with good results [3]. These programs seek to provide care to trauma patients in a planned sequence, assess compliance with established standards, and reduce variability of care in order to reduce costs while maintaining quality of care [4]. The TRISS and its derivations are an important tool in various techniques used to monitor quality of care for trauma patients, so the better its accuracy in estimating the probability of survival, the better its performance in trauma care quality improvement programs.

The adjustment of the survival probability equation coefficients according to the local population has been indicated despite the results of studies that show similar performance. Equations based on data from high-income countries do not seem to fit for use in low-income and middle-income countries [5–11].

The present study aimed to adjust the coefficients of three variations of the TRISS—NTRISS-like, TRISS SpO_2_, and NTRISS-like SpO_2_—for patients in the institutions in São Paulo (Brazil) and San Diego (USA) and compare the discriminatory ability of these equations when applied to different groups of trauma patients. These models have been evaluated in other study in which they have shown good accuracy (about 89.5%) and performance similar to other, previously published adjustments of the TRISS [12–21]. In these three variations, the Revised Trauma Score (RTS) was replaced in order to obtain the most adequate variables, according to their availability and which reflect the patient’s physiological condition.

The objective of the present study was to evaluate the accuracy of three variations of the TRISS in two different contexts: a level 1 university trauma center in San Diego County in the state of California, USA (a high-income country), and a university medical center located in the city of São Paulo, Brazil (a middle-income country).

## Methods

This was a retrospective study of trauma patients admitted to two centers: the Clinical Hospital of the College of Medicine of the University of São Paulo (HCFMUSP), a university medical center in São Paulo, Brazil (a middle-income country), and the Medical Center of the University of California at San Diego (UCSDMC), a level 1 university-based trauma center in the USA (a high-income country). The patients were admitted between January 1, 2006, and December 31, 2010. Victims of blunt or penetrating trauma aged 14 years or older were included in the study. Patients who were admitted to HCFMUSP and UCSDMC after 24 h of the traumatic event or transferred from other hospitals were excluded from the study. The traumatic events considered are listed in Chapter XX of the World Health Organization’s 10th International Classification of Diseases [22], excluding cases of hanging, suffocation, drowning or near drowning, poisoning, burns, and electrocution.

The data source for collecting information on patients in HCFMUSP was hospital records selected from a list containing the names and hospital record numbers of all patients hospitalized due to trauma. Patients who met the inclusion criteria were identified through the Hospital Information and Management System. Of the 3576 patients identified, 2416 were included in the study, making it a non-random sample composed of 67.6% of all cases.

At UCSDMC, the trauma patients admitted during the study period were identified in the institution’s database, which already contained all the necessary information for the research. In total, 8172 patients met the inclusion criteria, and access to information was obtained for all of them.

Thus, two matrix databases were generated: one for HCFMUSP, with 2416 patients, and one for UCSDMC, with 8172 patients. Three hundred patients were randomly selected from each of these databases, each record having all the information required to calculate the probability of survival rates, and these databases were designated the test databases. The information from other patients was grouped into derivation databases, forming two other databases: one for HCFMUSP, with 2116 patients, and one for UCSDMC, composed of 7872 patients.

Considering the disproportion between the number of patients in the HCFMUSP and the UCSDMC derivation database (2116 versus 7872), weight of 3.72 was given to each of the HCFMUSP patient in order that both database had the same weight in the coefficient derivation.

The test databases were used to evaluate the accuracy of the models, while the derivation databases were used to adjust the coefficients to the study population. The models applied were as follows: NTRISS-like (best motor response (BMR), systolic blood pressure (SBP), New Injury Severity Score (NISS), and age); TRISS SpO_2_ (Glasgow Coma Scale (GCS), SBP, peripheral oxygen saturation (SpO_2_), Injury Severity Score (ISS), and age); and NTRISS-like SpO_2_ (BMR, SBP, SpO_2_, NISS, and age). In the TRISS SpO_2_ and NTRISS-like SpO_2_, respiration rate was replaced with SpO_2_, considering that respiration rate is often not available in the primary assessment scenario, and observed changes in it cannot be directly related to respiratory function because of pain and psychological stress [23]. Furthermore, obtaining respiratory rate consumes time at a stage in treatment in which there is an urgent need for other approaches. In the NTRISS-like and NTRISS-like SpO_2_, the GCS was replaced by the item BMR on the scale, to enable the inclusion of intubated patients in analysis. Moreover, the NISS, which corrects the limitations of the ISS, was the anatomical index included in these variations of the TRISS [19, 24].

The demographic and clinical variables of patients were submitted to descriptive analysis, comparing the HCFMUSP and UCSDMC groups. The Pearson’s chi-square test was used for categorical variables, and ANOVA was used for continuous variables. The coefficients generated for all equations analyzed in this study were derived by means of logistic regression analysis. A 5% significance level was used for all tests.

Four equations were derived for each of the TRISS variations: two from the HCFMUSP and two from UCSDMC derivation database (one for blunt and other for penetrating trauma). These equations were validated in the HCFMUSP and UCSDMC test databases. The ROC curve and predictive ability of the models in each of the applications were evaluated. ROC curve performance was compared by DeLong’s Algorithm using *pROC* e *clinfun programs* [25–27].

## Results

The patient groups from HCFMUSP and UCSDMC were compared in relation to the characteristics presented in Table 1. The results showed statistically significant differences between the two groups in terms of all variables analyzed (*p* < 0.001).

**Table 1:** Descriptive statistics on age, gender, and clinical variables of patients from the HCFMUSP and UCSDMC. São Paulo-San Diego, 2006–2010

	Medical center	
	HCFMUSP	UCSDMC
	*N* (%)	*N* (%)
*Age (years), mean (SD)	40.0 (±17.9)	42.4 (±20.4)
*Gender		
Male	1922 (79.6)	5876 (71.9)
Female	494 (20.4)	2296 (28.1)
*Trauma mechanism		
Blunt	2141 (88.6)	7429 (90.9)
Penetrating	273 (11.3)	743 (9.1)
No information	2 (0.1)	–
*External causes of morbidity and mortality		
Land transport accidents	1425 (58.9)	3238 (39.6)
Falls	491 (20.3)	2712 (33.2)
Assaults	178 (7.4)	1730 (21.2)
Intentional self-harm	86 (3.6)	130 (1.6)
Events with undetermined intentions	129 (5.3)	76 (0.9)
Other	78 (3.3)	286 (3.5)
No information	29 (1.2)	–
*Emergency medical service		
No	211 (8.7)	149 (1.8)
Air	480 (19.9)	136 (1.7)
Basic life support	1183 (49.0)	34 (0.4)
Advanced life support	367 (15.2)	7803 (95.5)
Other	175 (7.2)	20 (0.2)
No information	–	30 (0.4)
*Surgical procedure		
Yes	1204 (49.8)	1532 (18.7)
No	1212 (50.2)	6640 (81.3)
*Admission in ICU		
Yes	1198 (49.6)	2934 (35.9)
No	1218 (50.4)	5238 (64.1)
*Survival		
Yes	1994 (82.6)	7968 (97.5)
No	422 (17.4)	204 (2.5)
*Glasgow Coma Scale		
3–8	742 (30.7)	427 (5.2)
9–12	230 (9.5)	318 (4.0)
13–15	1421 (58.8)	7350 (89.9)
No information	23 (1.0)	77 (0.9)
*Length of hospital stay (days), mean (SD)	10.5 (±18.0)	3.9 (±11.1)
*SBP (mmHg), mean (SD)	119.3 (±40.2)	137.9 (±27.5)
*RR (breaths/minute), mean (SD)	17.5 (±6.7)	18.2 (±4.9)
*SpO_2_, mean (SD)	94.1 (±10.2)	98.2 (±8.2)
*RTS, mean (SD)	6.3 (±2.3)	7.6 (±1.0)
*ISS, mean (SD)	13.7 (±10.1)	8.6 (±9.1)
*NISS, mean (SD)	18.1 (±12.2)	11.3 (±12.9)
*TRISS, mean (SD)	86.2 (±26.2)	96.3 (±12.4)

*ICU* intensive care unit, *SBP* systolic blood pressure, *RR* respiratory rate, *SpO*_*2*_ peripheral oxygen saturation, *RTS* Revised Trauma Score, *ISS* Injury Severity Score, *NISS* New Injury Severity Score, *TRISS* Trauma and Injury Severity Score

*p < 0.001

**Fig. 1:**
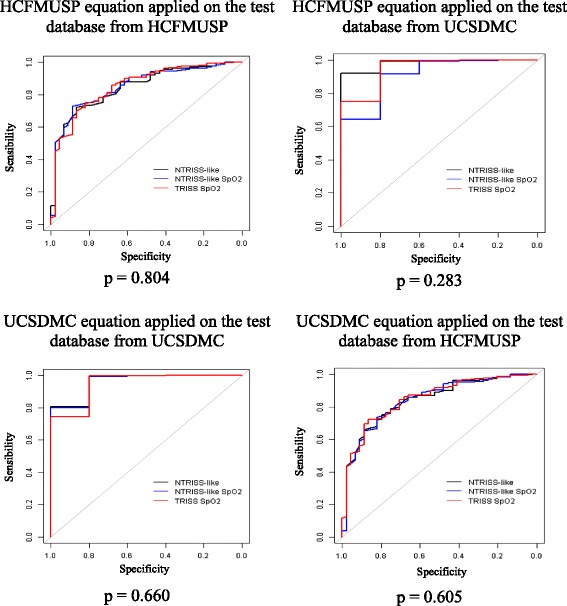
ROC curves from the equations, NTRISS-like, TRISS SpO_2_, and NTRISS-like SpO_2_ with coefficients derived from the HCFMUSP and UCSDMC derivation databases, applied on the different groups, for blunt and penetrating trauma. São Paulo-San Diego, 2006–2010

Coefficients were derived for each of the TRISS variations, as shown in Table 2. All of the equations generated were validated in the HCFMUSP and UCSDMC test databases, and their performance was compared, as shown in Fig. 1 and Table 3.

**Table 2:** Coefficients for NTRISS-like, TRISS SpO_2_, and NTRISS-like SpO_2_ derived from the HCFMUSP and UCSDMC derivation databases, for blunt and penetrating trauma. São Paulo-San Diego, 2006–2010

Coefficients	Blunt		Penetrating	
	HCFMUSP	UCSDMC	HCFMUSP	UCSDMC
NTRISS-like 1/(1 + *e* ^−*b*^), where *b* = *b*_0_ + *b*_1_ (BMR) + *b*_2_ (SBP) + *b*_3_ (NISS) + *b*_4_ (Age^a^)				
*b*_0_	−0.69833769	−0.23530206	−1.85975686	1.8503094
*b*_1_	0.51106398	0.62672859	0.65231593	0.3724069
*b*_2_	0.79908416	0.98770635	0.86044643	1.0745871
*b*_3_	−0.09919555	−0.07369974	−0.07452259	−0.1152176
*b*_4_	−1.58444558	−1.98269784	−0.31343883	−2.9135328
TRISS SpO_2_ 1/(1 + *e* ^−*b*^), where *b* = *b*_0_ + *b*_1_ (GCS) + *b*_2_ (SBP) + *b*_3_ (SpO_2_) + *b*_4_ (ISS) + *b*_5_ (Age)^a^				
*b*_0_	−0.68105065	0.07350818	0.4526497	−13.4424770
*b*_1_	0.59504995	0.96410432	0.8334234	1.1728932
*b*_2_	0.51134929	0.80528143	0.3053943	1.3872589
*b*_3_	0.29276785	0.10331305	1.1843363	2.6946416
*b*_4_	−0.08129884	−0.10089655	−0.2724244	−0.1016297
*b*_5_	−1.72272683	−2.12257955	−4.4829665	−3.0394087
NTRISS-like SpO_2_ 1/(1 + *e* ^−*b*^), where *b* = *b*_0_ + *b*_1_ (BMR) + *b*_2_ (SBP) + *b*_3_ (SpO_2_) + *b*_4_ (NISS) + *b*_5_ (Age) ^a^				
*b*_0_	−0.93885693	−1.61070692	−1.2399835	−9.4481884
*b*_1_	0.47015472	0.65710014	0.3143480	0.2461155
*b*_2_	0.52813273	0.88971953	0.7462718	1.6433308
*b*_3_	0.39359433	0.41984723	1.1698218	2.6394271
*b*_4_	−0.09505282	−0.07388783	−0.1278085	−0.1193648
*b*_5_	−1.70681763	−1.99504004	10.7638537	−3.8592826

*BMR* best motor response, *SBP* systolic blood pressure, *NISS* New Injury Severity Score, *GCS* Glasgow Coma Scale, *ISS* Injury Severity Score, *SpO*_*2*_ peripheral oxygen saturation

^a^Age—0 if <55 years old; 1 if ≥55 years old

**Table 3:** Predictive ability of the equations NTRISS-like, TRISS SpO_2_, and NTRISS-like SpO_2_, with coefficients derived from the HCFMUSP and UCSDMC derivation databases, applied to the different groups for blunt and penetrating trauma. São Paulo-San Diego, 2006–2010

	HCFMUSP equations validated on the HCFMUSP database				HCFMUSP equations validated on the UCSDMC database			
	Sens. (%)	Spec. (%)	AUC	95% CI	Sens. (%)	Spec. (%)	AUC	95% CI
NTRISS-like	72.3	86.4	0.848	0.791–0.905	92.2	100.0	0.982	0.952–1.000
TRISS SpO_2_	75.0	81.8	0.851	0.790–0.912	99.7	80.0	0.949	0.851–1.000
NTRISS-like SpO_2_	73.1	88.6	0.852	0.794–0.910	91.9	80.0	0.911	0.776–1.000
	UCSDMC equations validated on the UCSDMC database				UCSDMC equations validated on the HCFMUSP database			
	Sens. (%)	Spec. (%)	AUC	95% CI	Sens. (%)	Spec. (%)	AUC	95% CI
NTRISS-like	80.7	100.0	0.959	0.884–1	66.0	88.6	0.840	0.778–0.902
TRISS SpO_2_	99.7	80.0	0.948	0.847–1	77.3	86.4	0.849	0.789–0.908
NTRISS-like SpO_2_	80.0	100.0	0.958	0.880–1	78.8	81.8	0.843	0.781–0.906

*Sens* sensibility, *Spec* specificity, *AUC* area under curve

Figure 1 shows that there was no statistically significant difference (*p* > 0.05%) between the three models in each of the applications; therefore, the variations of TRISS had equivalent performance when applied to the same sample. Together with the information from Table 3, Fig. 1 shows that the performance of the equations was different when applied to patients in the origin institution and patients of the other institution: the equations derived from the HCFMUSP patients applied on the test database of the same hospital had an AUC of approximately 0.85 and in the San Diego group had an AUC between 0.911 and 0.982. Moreover, the application of equations derived in San Diego resulted in an AUC of about 0.95 when applied to the group of patients from the same institution and less than 0.85 on the group from HCFMUSP. The AUC was higher when the equations were applied at UCSDMC.

This may be because UCSDMC data included more low severity patients, which are easy population to predict outcome, than HCFMUSP. Patient characteristics are obviously different, so the predictive performance could be different. To exclude this possible bias, the models were derived and applied in patients with the same severity according to the ISS (< or ≥16). Table 4 shows that the models had similar AUC when applied to HCFMUSP and UCSDMC.

**Table 4:** Predictive ability of the equations NTRISS-like, TRISS SpO_2_, and NTRISS-like SpO_2_, with coefficients derived from the HCFMUSP and UCSDMC derivation databases, applied to the different groups for blunt and penetrating trauma, according to the severity by the Injury Severity Score (< or ≥16). São Paulo-San Diego, 2006–2010

	HCFMUSP equations validated on the HCFMUSP database				HCFMUSP equations validated on the UCSDMC database			
	ISS <16		ISS ≥16		ISS <16		ISS ≥16	
	AUC	95% CI	AUC	95% CI	AUC	95% CI	AUC	95% CI
NTRISS-like	0.908	0.878–0.938	0.867	0.836–0.898	0.901	0.868–0.933	0.868	0.837–0.898
TRISS SpO_2_	0.871	0.834–0.908	0.846	0.813–0.878	0.882	0.847–0.917	0.849	0.818–0.881
NTRISS-like SpO_2_	0.892	0.860–0.923	0.862	0.830–0.894	0.887	0.854–0.920	0.861	0.829–0.894
	UCSDMC equations validated on the UCSDMC database				UCSDMC equations validated on the HCFMUSP database			
	ISS <16		ISS ≥16		ISS <16		ISS ≥16	
	AUC	95% CI	AUC	95% CI	AUC	95% CI	AUC	95% CI
NTRISS-like	0.909	0.863–0.954	0.936	0.913–0.958	0.899	0.850–0.948	0.936	0.913–0.958
TRISS SpO_2_	0.911	0.861–0.961	0.921	0.894–0.948	0.894	0.827–0.960	0.911	0.883–0.940
NTRISS-like SpO_2_	0.885	0.817–0.954	0.926	0.901–0.951	0.870	0.796–0.944	0.926	0.900–0.951

*ISS* Injury Severity Score, *AUC* area under curve

## Discussion

The equations derived with data from HCFMUSP and that derived with data from UCSDMC showed greater accuracy when applied to the San Diego population than to patients from São Paulo. However, deriving and applying the models (HCFMUSP and UCSDMC) in these two populations classified by severity, equations presented similar accuracy both in São Paulo and in San Diego.

The application of the equations to patients from HCFMUSP with coefficients adjusted to this population did not increase the accuracy of the models (AUC between 0.848 and 0.852), since the equations adjusted to patients from UCSDMC had similar performance in this hospital (AUC between 0.840 and 0.849). This result raises uncertainties about the importance of adjustments of survival probability rates to the local realities where they are applied. This uncertainty is also reinforced by the results of other studies that have shown equivalent or worse performance of the TRISS after these coefficient adjustments [1, 6, 28–32].

The accuracy of the models was similar to the same test population, and all the equations presented better performance when validated for patients from UCSDMC, regardless of the group in which they were derived. Considerations should be made about these observations.

The characteristics of the population in which the equations were validated have contributed to this result, since the performance was better in the UCSDMC group. The subjects in the groups differed significantly in all statistical comparisons. Most of the patients from the two institutions received prehospital care. In HCFMUSP, there was a predominance of basic life support, while in UCSDMC, there was a predominance of advanced life support (ALS). Considering the severity of HCFMUSP, it is desirable that they receive ALS prehospital care. The length of hospital stay, frequency of admission to ICU, and surgical procedures were significantly higher among those admitted to HCFMUSP. As regards severity, the São Paulo patients were more severely traumatized than those in San Diego. Mortality in HCFMUSP was significantly greater than that in UCSDMC.

UCSDMC data included more low severity patients, which are easy population to predict outcome, than HCFMUSP. As the characteristics are obviously different, so the predictive performance could be different. When the accuracy was evaluated considering the trauma severity by ISS, the results showed similar AUC for both HCFMUSP and UCSDMC equations, regardless of the group they were applied.

Evaluating the AUC of the applied equations in the HCFMUSP population, five in six presented better performance when the equations were derived and applied in patients with the same severity (0.862–0.926) than in general population (0.840–0.852). These findings were not found in the population of San Diego, who presented smaller AUC when analyzed by equations derived and applied in similar patients for severity (0.849–0.936 versus 0.911–0.982).

In addition to severity, other factors should be considered. The two patient groups were inserted into different social, political, and economic realities. Additionally, the group that was more severely traumatized was cared for in a hospital that is a reference center for trauma care in an emergency care network that is in an organizational phase (HCFMUSP). The other group of patients, who were less severely traumatized, was cared for in a level I trauma care center (UCSDMC), inserted into a consolidated system of care for trauma victims with established prevention programs.

The UCSDMC is part of a consolidated and well-established trauma system and has a low mortality rate. A well-structured care system allows greater uniformity and predictability of care provided to victims of trauma, with less chance of non-adherence to care protocols [33]. In addition, UCSDMC works with an activation protocol for the trauma team, which prepares to receive patients prior to arrival at the hospital. Better results have been shown for individuals who are treated in accordance with this protocol [16], which has certain advantages: it enables better communication between the emergency and hospital care teams; it allows prior availability of resources that patients might need; it involves teams in order to evaluate patients as a whole and properly prioritize investigations; and it creates a suitable setting for trauma education and research [16].

In HCFMUSP, trauma patients receive care in the emergency room and are treated by the medical staff of emergency general surgery, who are responsible for treating all surgical emergencies admitted to this sector. Other health care professionals, including nurses, do not have specific training for trauma. The mortality rate in this institution was 17.5%, but mortality between 1 and 38% was found in the literature [34].

Studies have linked improvements in the results of care for trauma patients with regionalized trauma systems with trauma center designation [13, 16]. Well-structured trauma care systems enable better distribution of resources, respecting the “golden hour” and offering all severely traumatized patients’ complexity of treatment according to need. Patients with similar needs receive treatment according to specific protocols based on those needs.

There is an increasingly critical need for the creation of a Brazilian system of trauma care, not only to improve trauma records but also to strengthen quality improvement programs, enhance the results of trauma care, decrease mortality rates, and allow the return of these individuals to society with quality of life. The training of professionals deserves special attention in this process. Some trauma training programs of the American College of Surgeons Committee on Trauma, Society of Trauma Nurses, and National Association of Emergency Medical Technicians are available nationally.

Access to the highest level of care, injury prevention strategies, system-wide quality assurance, population-based surveillance of injury-related problems, disaster preparedness programs, financial viability, and integration with the existing health system are critical elements of an integrated system and should be addressed to an effective trauma system in Brazil.

This study presented some limitations. The sample of patients from HCFMUSP was not random, because access to the records was according to their availability. However, according to information from persons responsible for the division of medical records, the search for the records requested was casual. We used the 2005 version 2008 update for coding the injuries of the patients from HCFMUSP and the 1998 version for patients from UCSDMC. This difference occurred because in the data from HCFMUSP, the injuries were coded recently and the current AIS version was used. In San Diego, the AIS codes were established at the same time as the inclusion of records in the database, and the records were coded according to the 1998 version. In one study in Australia comparing the use of these two versions in patients with ISS >15, the ISS presented better performance when calculated with the AIS 2008; however, there was no difference in the performance of the NISS, TRISS, and NTRISS with the use of the 1998 and 2008 versions of AIS [35].

## Conclusions

Survival probability models showed better performance when applied in data collected in the HIC, regardless the country they were derived—high- or middle-income country. The severity is an important factor to consider when using non-adjusted survival probability models for the local population—adjusted models for severely traumatized patients better predict survival probability in less severely traumatized populations, as adjusted survival probability models for the local population. More severely traumatized populations benefit when survival probability models were adjusted according to the trauma patients’ severity.
